# Supplementary material for: Engineering Bio-Adhesives Based on Protein–Polysaccharide Phase Separation
Source: Int J Mol Sci. 2022 Sep 1;23(17):9987. doi: 10.3390/ijms23179987 (PMC9456018; doi:10.3390/ijms23179987)
Supplement: Supplementary file 1 [file ijms-23-09987-s001.zip › ijms-1880935-supplementary.pdf]

International Journal of
Molecular Sciences

MDPI

Supplementary Information

Engineering bio-adhesives based on protein-polysaccharide phase separation

Zoobia Bashir†, Wenting Yu†, Zhengyu Xu, Yiran Li, Ying Li*, Yi Cao and Bin Xue*

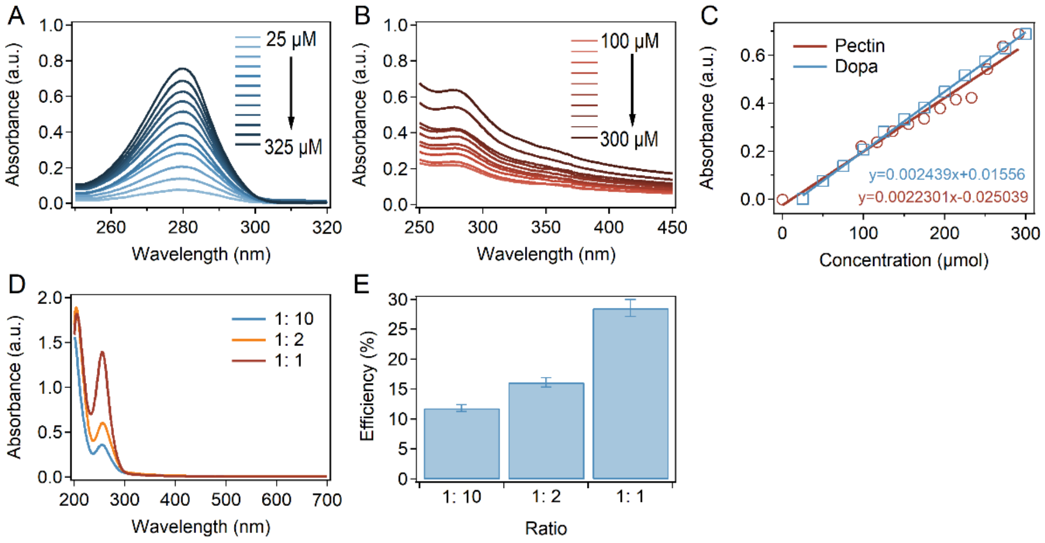

Figure S1. Determination of the reaction efficiency between dopa and pectin. (A) UV absorbance of Dopa at different concentrations (25 μM - 325 μM). (B) UV absorbance of pectin at different concentrations (100 μM - 300 μM). (C) Calibration curve of the dopa and pectin concentrations and OD_{212nm}. (D) UV spectra of pectin-dopa at different mass ratios of dopa and pectin (1: 10, 1: 2 and 1: 1). (E) Reaction efficiency of dopa and pectin. Error bars represent SD.

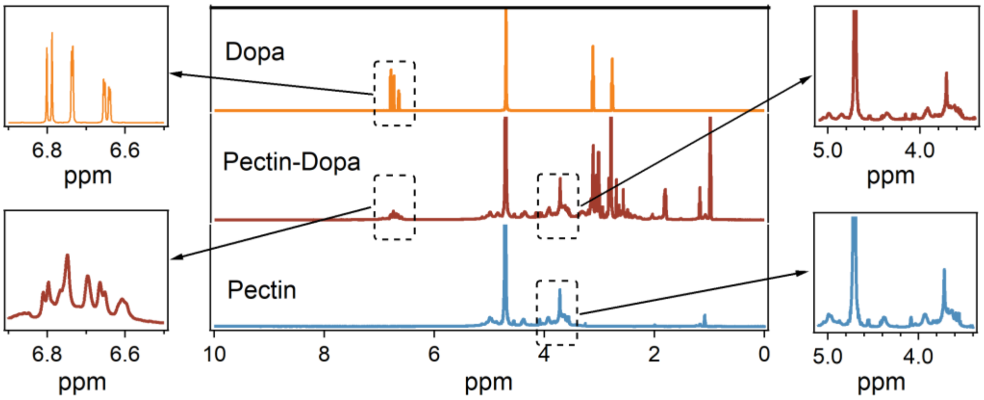

Figure S2. H Nuclear Magnetic Resonance (^{1}H-NMR) spectroscopy of dopa, pectin and pectin-dopa

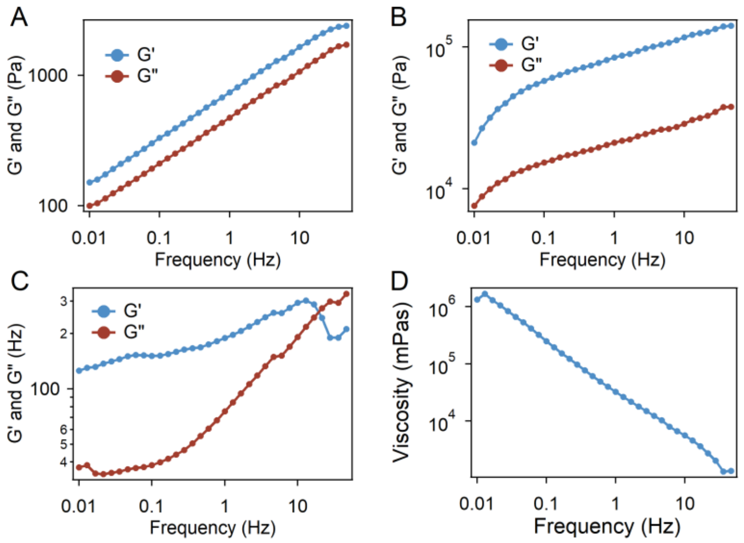

Figure S3. Rheological properties of the bio-glues. (A-C) G′ and G″ of pectin-dopa (A), P-L (B) and PD-L (C) bio-glue with ion measured in a frequency sweep experiment (from 0.01 to 90 Hz, 1% strain) at room temperature. (D) Viscosity measurement of PD-L bio-glue at 20 °C.

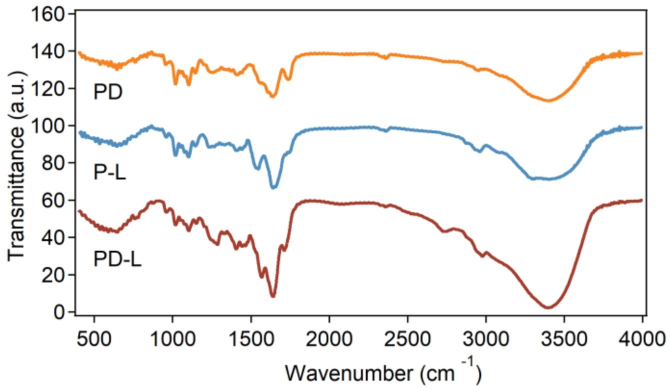

Figure S4. Fourier transform infrared (FT-IR) spectroscopy of the PD, P-L and PD-L bio-glues.

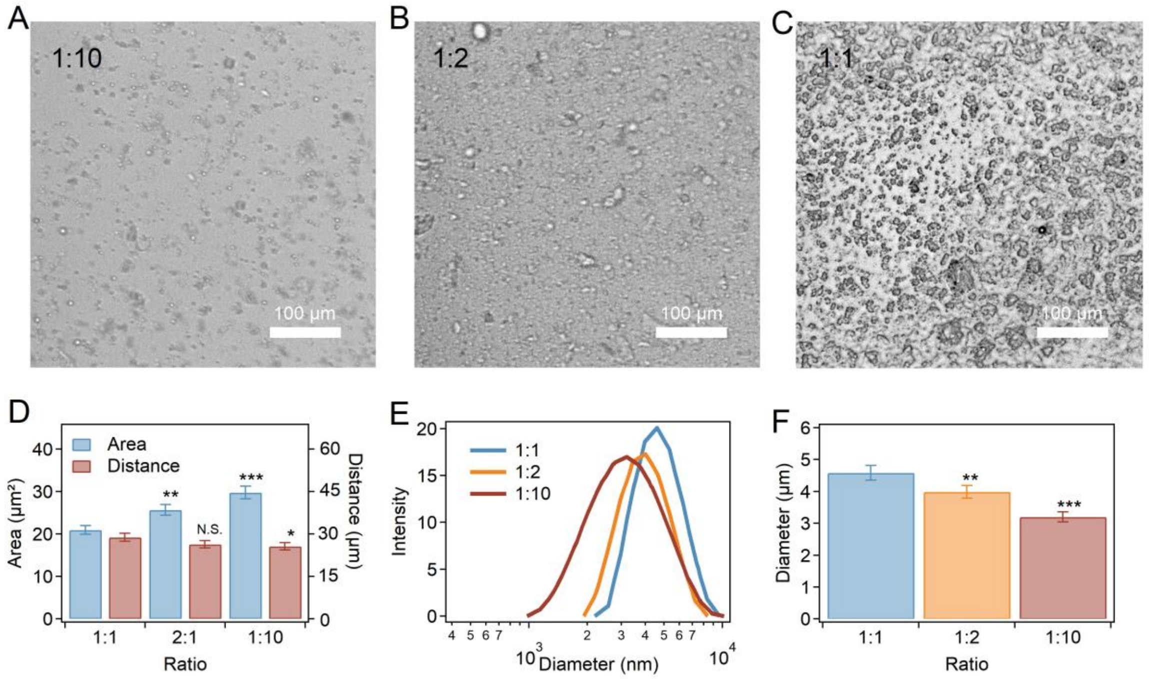

Figure S5. Phase separation of PD-L bio-glues at different synthesis ratios of dopa and pectin. (A-C) Micrographs of the mixture of PD-L bio-glue prepared using pectin-dopa synthesized at different mass ratios of dopa and pectin. A: 1: 10; B: 1: 2; C: 1: 1. (D) Average distance between the adjacent coacervates and average area of the coacervates in PD-L bio-glue at different synthesis ratios of dopa and pectin. Error bars represent SD. (E-F) Diameter distribution (E) and average diameter of coacervates (F) in PD-L bio-glues at different synthesis ratios of dopa and pectin measured using DLS. Error bars represent SD. Asterisks denote statistical significance compared with bio-glue at a ratio of 1: 1 ($P>0.05$: N.S.; $P < 0.05$: *; $P < 0.01$: **; $P < 0.001$: ***).

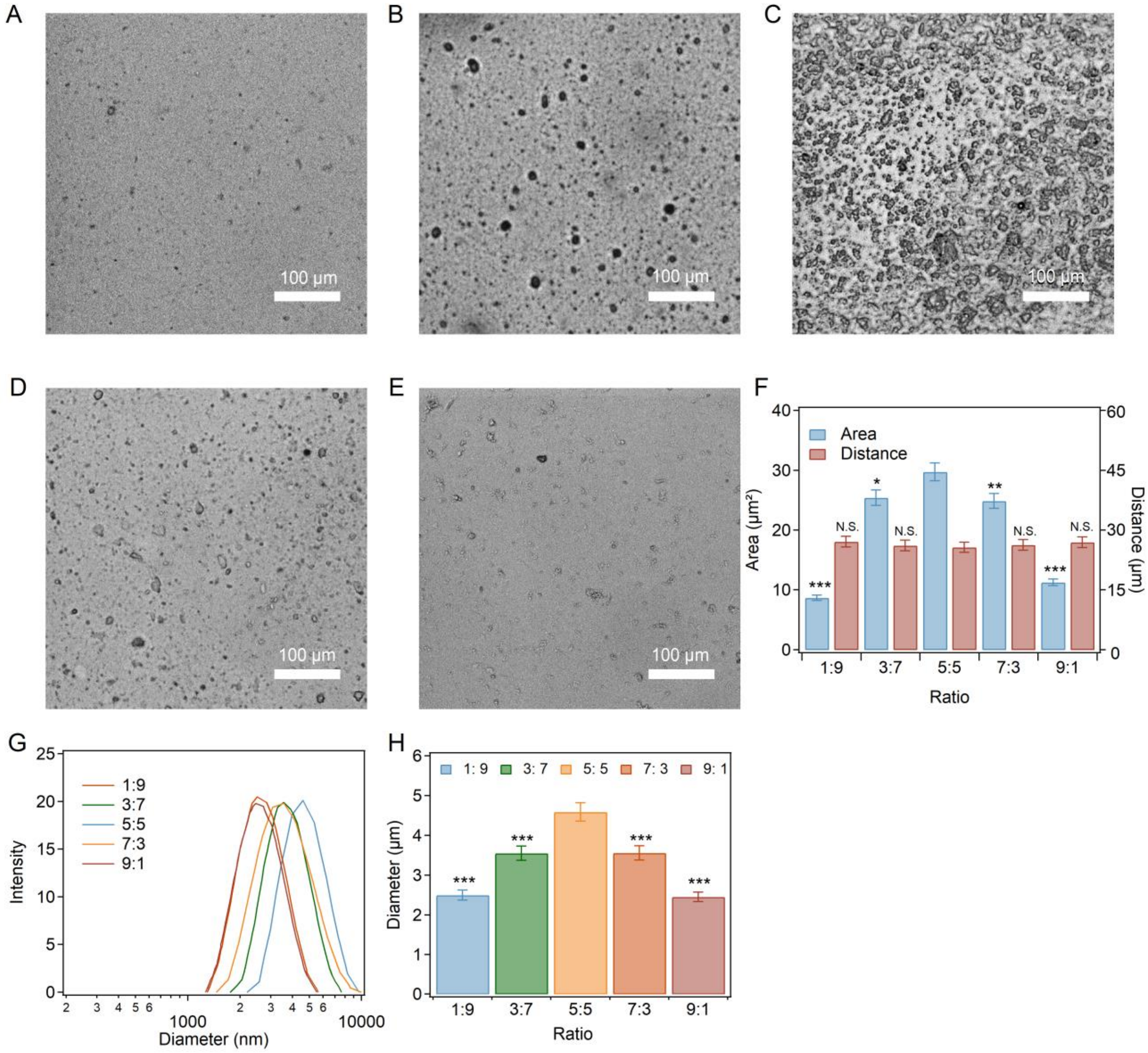

Figure S6. Phase separation of pectin-dopa and β-lactoglobulin at different mass ratios of pectin-dopa and β-lactoglobulin. (A-E) Micrographs of the mixture of pectin-dopa and β-lactoglobulin in PBS (10 mM, pH = 7.4) at different pectin-dopa: β-lactoglobulin ratios (1: 9, 3: 7, 5: 5, 7: 3 and 9: 1). (F) Average distance between the adjacent coacervates and average area of the coacervates at different pectin-dopa: β-lactoglobulin ratios (1: 9, 3: 7, 5: 5, 7: 3 and 9: 1). Error bars represent SD. (G) Coacervate size distribution (G) and summarized diameter of coacervates (H) in the mixture of pectin-dopa and β-lactoglobulin at different pectin-dopa: β-lactoglobulin ratios (1: 9, 3: 7, 5: 5, 7: 3 and 9: 1). Error bars represent SD. Error bars represent SD. Asterisks denote statistical significance compared with bio-glue at a ratio of 5: 5 (P>0.05: N.S.; P < 0.05: *; P < 0.01: **; P < 0.001: ***).

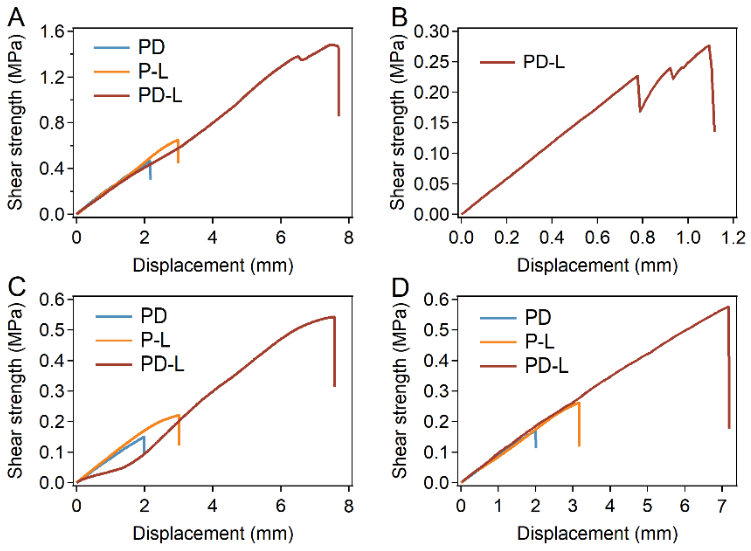

Figure S7. Typical force-displacement curves in shear strength measurements using different glues. (A-B) Typical force-displacement curves recorded in the shear strength measurements for long-term (A) and short-term (B) skin-skin adhesion using different glues. (C) Typical force-displacement curves recorded in the shear strength measurements for long-term glass-glass adhesion. (D) Typical force-displacement curves recorded in the shear strength measurements for long-term glass-skin adhesion.

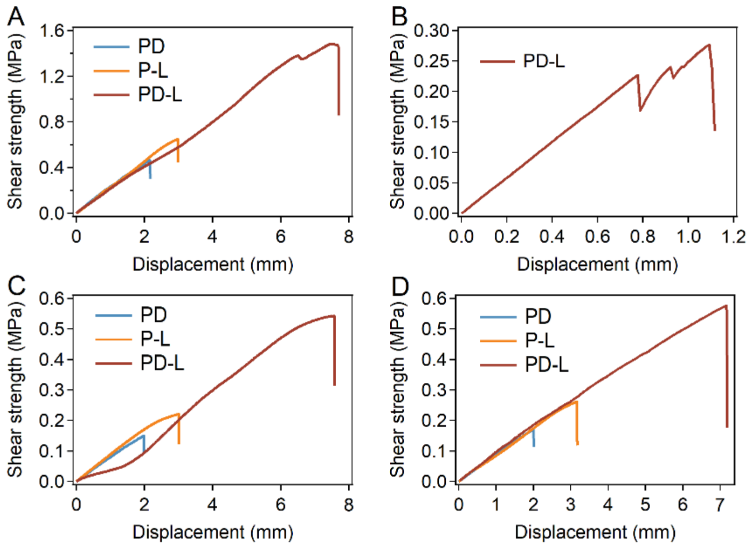

Figure S8. Typical force-displacement curves in tensile strength measurements using different glues. (A-B) Typical force-displacement curves recorded in the tensile strength measurements for long-term (A) and short-term (B) skin-skin adhesion. (C) Typical force-displacement curves recorded in the tensile strength measurements for long-term glass-glass adhesion. (D) Typical force-displacement curves recorded in the tensile strength measurements for long-term glass-skin adhesion.

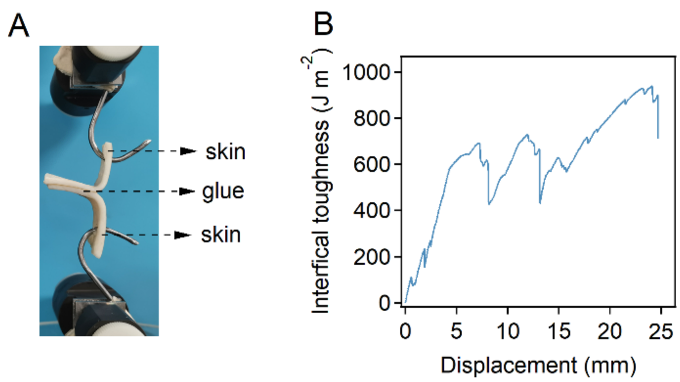

Figure S9. Interfacial toughness determination of the adhesion for porcine skin using PD-L glue. (A-B) Typical image (A) and force-displacement curve (B) recorded in the test of interfacial toughness.

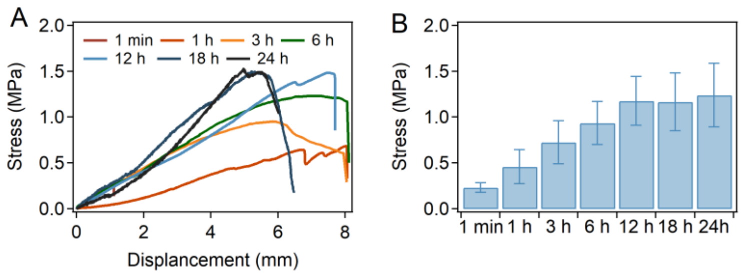

Figure S10. Adhesion strength of PD-L bio-glue at different oxidation times. (A) Typical force–displacement curves recorded in the lap shear experiment for the porcine skin adhesion using PD-L bio-glue after curing for different times. (B) Adhesion strength for the porcine skin using PD-L bio-glue after curing for different times. Error bars represent SD.

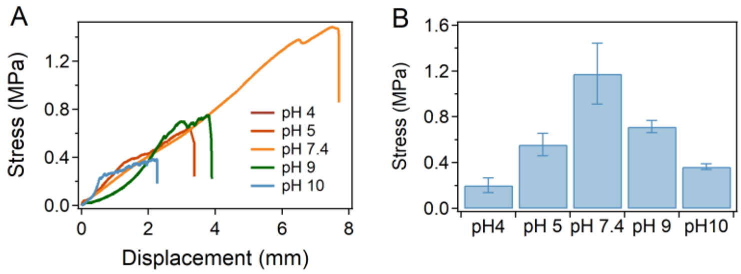

Figure S11. Adhesion strength of PD-L bio-glue at different pH. (A) Typical force–displacement curves recorded in the lap shear experiments for the long-term porcine skin adhesion using PD-L bio-glue prepared using PBS at different pH (4.0, 5.0., 7.4, 9.0 and 10.0). (B) Adhesion strength for the long-term porcine skin adhesion using PD-L bio-glue prepared using PBS at different pH. Error bars represent SD.

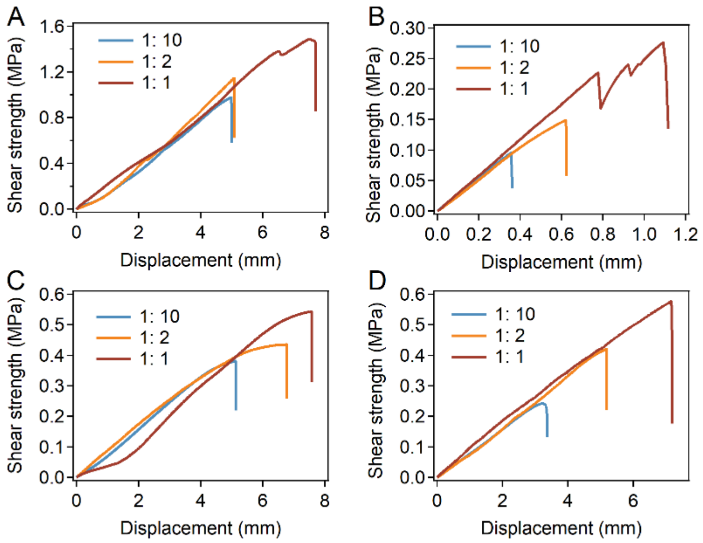

Figure S12. Adhesion performance of the bio-glue at different synthesis ratios of dopa and pectin (1: 10, 1: 2 and 1: 1). (A-B) Typical force-displacement curves recorded in the shear strength measurements for long-term (A) and short-term (B) skin-skin adhesion at different synthesis ratios of dopa and pectin. (C) Typical force-displacement curves recorded in the shear strength measurements for long-term glass-glass adhesion at different synthesis ratios of dopa and pectin. (D) Typical force-displacement curves recorded in the shear strength measurements for long-term skin-glass adhesion at different synthesis ratios of dopa and pectin.

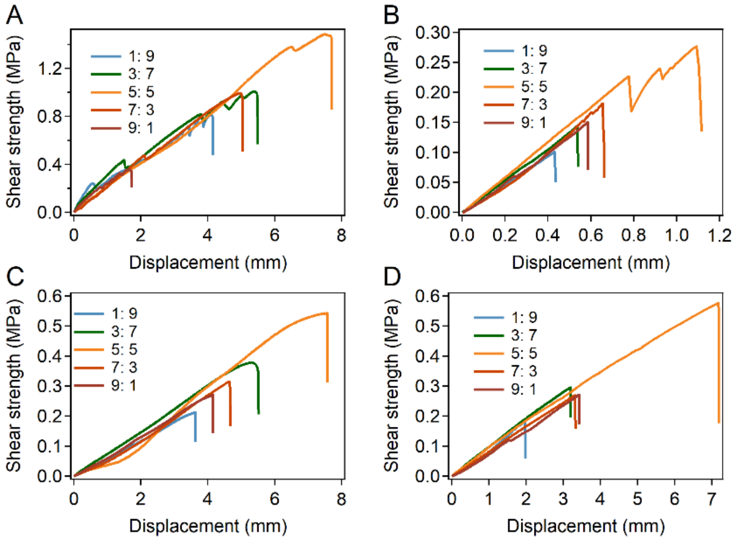

Figure S13. Adhesion performance of bio-glue at different pectin-dopa:β-lactoglobulin ratios. (A-B) Typical force-displacement curves recorded in the shear strength measurements for long-term (A) and short-term (B) skin-skin adhesion at different pectin-dopa: β-lactoglobulin ratios (1: 9, 3: 7, 5: 5, 7: 3 and 9: 1). (C) Typical force-displacement curves recorded in the shear strength measurements for long-term glass-glass adhesion at different pectin-dopa:β-lactoglobulin ratios (1: 9, 3: 7, 5: 5, 7: 3 and 9: 1). (D) Typical force-displacement curves recorded in the shear strength measurements for long-term skin-glass adhesion at different pectin-dopa: β-lactoglobulin ratios (1: 9, 3: 7, 5: 5, 7: 3 and 9: 1).

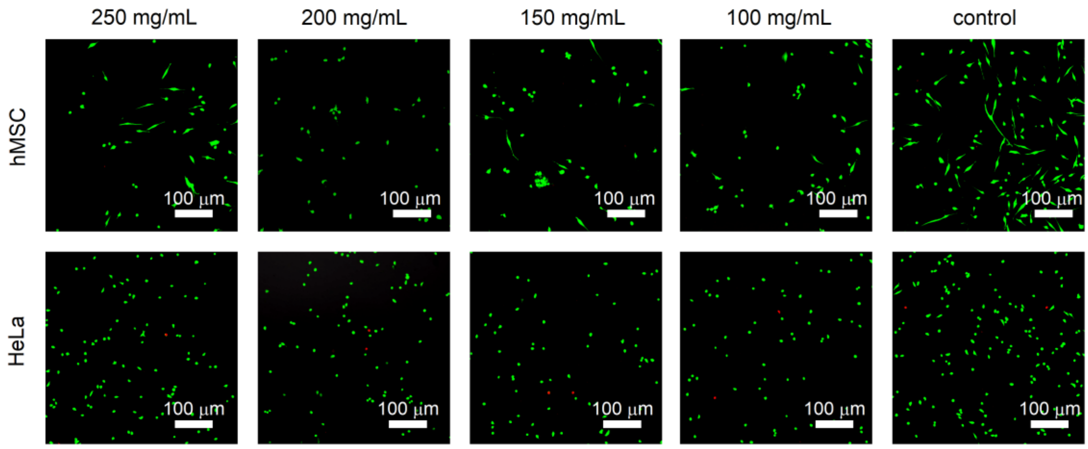

Figure S14. Fluorescence microscopic images of HeLa (left) and hMSC (right) cells cultured in the presence of bio-glue (50, 100, 150, 200 and 250 mg mL^{-1} and control group. The living and dead cells were stained with a live/dead assay (Calcein-AM/PI Double Staining Kit) after 24 h of culture.
